# Genetic changes associated with the temporal shift in invasive non-typhoidal *Salmonella* serovars in Bamako Mali

**DOI:** 10.1371/journal.pntd.0007297

**Published:** 2019-06-06

**Authors:** Kristin Bornstein, Sharon M. Tennant, Tracy H. Hazen, John D. Sorkin, Milagritos D. Tapia, Samba O. Sow, Uma Onwuchekwa, Myron M. Levine, David A. Rasko

**Affiliations:** 1 Center for Vaccine Development and Institute for Global Health, University of Maryland School of Medicine, Baltimore, MD, United States of America; 2 Department of Epidemiology & Public Health, University of Maryland School of Medicine, Baltimore, MD, United States of America; 3 ICF, Lee Highway, Fairfax, Virginia, United States of America; 4 Baltimore VA Medical Center Geriatric Research, Education, and Clinical Center, and Department of Medicine, University of Maryland School of Medicine, Baltimore, MD, United States of America; 5 Institute for Genome Sciences, Department of Microbiology and Immunology, University of Maryland School of Medicine, Baltimore, MD, United States of America; 6 Department of Pediatrics, University of Maryland School of Medicine, Baltimore, MD, United States of America; 7 Centre pour le Développement des Vaccins, Mali (CVD-Mali), Bamako, Mali, Africa; The University of Sheffield, UNITED KINGDOM

## Abstract

**Background:**

Invasive non-typhoidal *Salmonella* (iNTS) serovars *S*. Typhimurium and *S*. Enteritidis are major etiologic agents of invasive bacterial disease among infants and young children in sub-Saharan Africa, including in Mali. Early studies of iNTS serovars in several countries indicated that *S*. Typhimurium was more prevalent than *S*. Enteritidis, including in Mali before 2008. We investigated genomic and associated phenotypic changes associated with an increase in the relative proportion of iNTS caused by *S*. Enteritidis versus *S*. Typhimurium in Bamako, Mali, during the period 2002–2012.

**Methodology/Principal findings:**

Comparative genomics studies identified homologs of tetracycline resistance and arsenic utilization genes that were associated with the temporal shift of serovars causing iNTS shift, along with several hypothetical proteins. These findings, validated through PCR screening and phenotypic assays, provide initial steps towards characterizing the genomic changes consequent to unknown evolutionary pressures associated with the shift in serovar prevalence.

**Conclusions/Significance:**

This work identified a shift to *S*. Enteritidis from the more classic *S*. Typhimurium, associated with iNTS in Bamako, Mali, during the period 2002–2012. This type of shift in underlying iNTS pathogens are of great importance to pediatric public health in endemic regions of sub-Saharan Africa. Additionally, this work demonstrates the utility of combining epidemiologic data, whole genome sequencing, and functional characterization in the laboratory to identify and characterize genomic changes in the isolates that may be involved with the observed shift in circulating iNTS agents.

## Author summary

Much remains unknown about the mode of transmission of iNTS or the reservoirs of infection. As such, insight into potential selective pressures on underlying serovars bears importance to public health. Longitudinal studies over the years 2002–2012 identified a shift in the proportion of invasive non-typhoidal *Salmonella* (iNTS) disease caused by *S*. Typhimurium or *S*. Enteritidis in Bamako, Mali. Since *S*. Enteritidis exhibits a higher rate of fatal cases among the pediatric population than *S*. Typhimurium, it is important to understand what led to the increased proportion of cases from this serovar. This study examined the genetic changes in *S*. Enteritidis associated with this serovar shift through comparative genomics and laboratory findings. Through these methods, genes related to tetracycline resistance and arsenic catabolism were associated with the shift in serovars. These findings represent preliminary steps in investigating this underlying shift and determining the long-term repercussions of these changes to the epidemiologic profile of iNTS disease.

## Introduction

*Salmonella enterica* is a bacterial pathogen that includes over 2500 serological variants (serovars) and is estimated to cause more than 1.3 billion cases of clinical illness annually worldwide [1]. Non-typhoidal *Salmonella* (NTS) serovars such as *S*. Enteritidis and *S*. Typhimurium, while mainly limited to gastrointestinal illness in industrialized nations [2,3], have been found to cause severe invasive bacterial disease in sub-Saharan Africa [4–6] typically unaccompanied by or preceded by diarrhea. Children from 6–12 months of age suffer the greatest incidence of invasive disease, and case fatality rates for *S*. Enteritidis disease are high (20–28%) in infants and young children in Africa [7]. In part, the differences in clinical presentation of NTS disease in pediatric populations in sub-Saharan Africa versus in industrialized countries may be due to immunosuppression, malnutrition, intensity of previous antigenic exposure, or co-infections, particularly malaria [5,8–10]. However, there are also differences in the inherent pathogenicity of strains of non-typhoidal *Salmonella*, in addition to the host and infection-related factors [11–16]. For example, ~95% of the invasive *S*. Typhimurium strains in Africa are multi-locus sequence type 313 (ST313), a genotype unique to that continent that has undergone extensive genomic degradation associated with host-adaptation [11,13] and which is different from the ST19 strains that predominate outside Africa [11,17].

*S*. Typhimurium has been identified as the leading cause of invasive NTS (iNTS) disease in many countries in sub-Saharan Africa [14], although in some countries *S*. Enteritidis was the predominant serovar [18]. Systematic surveillance undertaken by the Center for Vaccine Development of Mali (CVD-Mali) and by the Center for Vaccine Development at the Hôpital Gabriel Touré (HGT) in Mali revealed a sizable burden of iNTS disease [19]. This surveillance was initiated in 2002 to quantify the burden of invasive bacterial disease caused by *Haemophilus influenzae* type b (Hib) and *Streptococcus pneumoniae* (pneumococcus) and to assess the need for introduction of vaccines to prevent disease caused by those pathogens, which were responsible for most cases of pediatric bacteremia. Although not originally designed to do so, review of the surveillance data identified NTS serovars Typhimurium and Enteritidis as common causes of severe invasive disease. Moreover, after routine infant immunization with Hib and pneumococcal conjugate vaccines was implemented and those two infections were controlled, NTS emerged as the predominant cause of invasive bacterial disease in infants and young children in Mali [19]. The quality and duration of the systematic surveillance at HGT facilitated identification of a shift in iNTS epidemiology around 2008 [12]. An increased relative proportion of iNTS disease due to *S*. Enteritidis was detected along with a concurrent decrease in the prevalence of *S*. Typhimurium after 2008 [19]. From the iNTS isolates included in the epidemiologic investigation by Tapia et al. [19], a subset of 42 *S*. Enteritidis isolates, selected as a stratified sample by year of isolation, were included in a phylogenetic analysis examining global lineages of *S*. Enteritidis conducted by Feasey et al. [15]. Examination of the genomic phylogeny suggested that the newly circulating *S*. Enteritidis isolates collected from Mali (2008–2012) are phylogenomically distinct from the 2002–2008 isolates. While periodic *Salmonella* serovar shifts have been observed elsewhere in the world [20], the genotypic characteristics and environmental pressures that lead to such serovar shifts are not well understood and have not been investigated in detail, especially using whole genome sequencing.

Although data on the characterization of major genomic elements and evolutionary pressures on non-typhoidal *Salmonella* including *S*. Enteritidis are limited, studies by Nuccio and Baumler [21,22] have identified genomic signatures associated with increased growth within the host gastrointestinal tract and increased gut inflammation. These signatures are identified with NTS strains associated with gastroenteritis, while the genome of NTS strains associated with invasive disease is thought to be degraded [12]. Feasey et al. [15] examined global lineages of *S*. Enteritidis, including the isolates in this study, and identified two clades of *S*. Enteritidis that have become prominent in Africa. Feasey et al. [15] also established that the *S*. Enteritidis clades exhibit similar host-adapted genomic degradation as seen with *S*. Typhi, *S*. Paratyphi A, and *S*. Typhimurium. None of the previous studies investigated genomic changes in *S*. Enteritidis within a discrete population, such as the pediatric population of Bamako. Our current study was undertaken to identify and characterize genomic markers in *S*. Enteritidis isolates that are representative of the clade that emerged as the predominant serovar of iNTS disease in the pediatric population in Bamako, Mali during the time period since systematic surveillance was established. This work demonstrates the utility of combining epidemiological principles, whole genome sequencing, and functional characterization in the laboratory to identify and characterize genomic changes in the isolates that may be responsible for the observed shift in the iNTS serovars.

## Methods

### Sample acquisition

Isolates included in this study were collected by local hospital staff from the blood or spinal fluid of patients during hospital admission at the HGT, as reported by Tapia et al. [19]. The serovar of these isolates was confirmed through multiplex polymerase chain reaction (PCR) assay and agglutination with *Salmonella* antiserum. The 42 isolates submitted for whole-genome sequencing (S1 Table), as described by Feasey et al. [15] and utilized for the *in silico* analysis in our study, had their taxonomy verified by culture on selective *Salmonella*-*Shigella* agar, positive O (D1) agglutination from cultures grown on Trypticase Soy Agar, and positive H agglutination (with antisera against g and m Phase 1 H antigens) from cultures grown on 0.6% agar swarm medium.

### Establishing a temporal breakpoint in the relative abundance of serovars

We examined a phylogenetic tree based on the core genome of the same 42 *S*. Enteritidis isolates examined by Feasey et al. [15]. The proportion of misclassified isolates was determined for each year and at each branch of the phylogenetic tree, with year of isolation determined by the seasonal trends in weather (June-July) [19]. Isolates were considered misclassified when they were collected from one side of the temporal breakpoint, but appeared in a branch that consisted mainly of isolates from the other side of the breakpoint. The branches of the tree were manually rotated at each node to determine the potential breakpoint year to minimize the number of misclassifications across the entire tree. Each year was tested as a potential breakpoint and the year which resulted in the lowest proportion of misclassifications across the major branches of the tree was ultimately selected. This temporal breakpoint was then compared to the epidemiologic data reported from the HGT [19] and the global phylogenetic analysis performed by Feasey et al. [15].

### Bioinformatic genetic analysis

The whole genome sequences of the 42 *S*. Enteritidis isolates (sequenced by the Wellcome Trust Sanger Institute) were assembled and annotated using the Velvet assembly through the CloVR pipeline, release version 1.0-RC5 [23] at the Institute for Genome Sciences (IGS) at the School of Medicine, University of Maryland, Baltimore. The sequence of each isolate was analyzed for protein encoding genes using a protein-coding gene prediction program, Prodigal (Prokaryotic Dynamic Programming Genefinding Algorithm) [24].

Once all the putative genes had been identified, BLAST Score Ratios (BSRs) [25] were calculated for each putative gene in each examined isolate using Large Scale BLAST Ratio Analysis (LS-BSR) [26]. The protein-coding genes in all of the genomes were predicted using Prodigal and were clustered by >90% similarity using uclust. BSR is calculated as the bit score of a gene detected in a genome divided by the bit score of the gene compared to itself, resulting in a scale normalized from 0 to 1. A BSR of ≥ 0.8 indicated a gene was present in a genome with significant similarity, a BSR ≤ 0.4 indicated a gene was absent, and genes identified with BSR values <0.8 but ≥ 0.4 were considered present with divergent similarity. The predicted protein-coding genes that were present (BSR≥ 0.8) in all isolates were removed from further analysis, as they constituted the conserved core genome, resulting in a list of 1,220 putative genes with variable presence among the included isolates (S1 Table). To identify genes associated with the genetic shift in *S*. Enteritidis, methods similar to those described by Sahl et al. [26] were used. Briefly, significant differences in the average BSRs before and after the 2008 breakpoint for each gene were identified using Student’s t-tests resulting in a p<0.01 (Welch’s method was used to account for unequal variances). Genes significantly associated with either the pre- or post-2008 time periods were then verified by manual BLAST searches in each genome. This verification was performed to ensure that any differences in BSR were due to a true association with the 2008 cut point, and were not missing from the draft genome assemblies as an artifact of the sequencing and assembly.

### Phylogenomic analysis

The genomes of the isolates in this study were compared by whole-genome phylogenomic analysis as previously described [15]. The genomes were aligned using Mugsy [27] and homologous blocks were concatenated using the bx-python toolkit [28]. The columns that contained one or more gaps were removed using Mothur [29]. The concatenated regions from each genome were used to construct a maximum-likelihood phylogeny with 100 bootstrap replicates using RAxML v7.2.8 [30] that was visualized using FigTree v1.4.2 [31]. Isolates that were isolated before or during 2008 are colored black and those post-2008 are colored blue.

### Sample size and selection

From surveillance through June 30^th^, 2012, the isolates from 299 individuals identified to have laboratory-confirmed invasive infections caused by *S*. Enteritidis were collected. Of the 103 available isolates obtained before or during 2008, 23 were sequenced using the Illumina platform. Nine of these 23 (39%) exhibited one of the putative genes identified as significantly associated with the shift in *S*. Enteritidis. This gene (centroid_210035_1) possessed the weakest level of statistical significance (p = 0.0009) among the putative genes associated with the serovar shift. Of the 196 isolates obtained from after 2008, 19 were sequenced, with 16 exhibiting the present allele of the same gene (84%). By focusing on this least significant associated gene, we used these prevalence rates to estimate conservative sample size calculations for examining the distribution of this, and more significantly associated genes across other available isolates based on a binomial distribution across two samples. A two-sided sample size analysis indicated that screening 72 additional samples [36 from each group, pre- and post-2008) would provide 80% power by PCR to identify genetic changes across all available isolates. Simple random sampling of all isolates collected before and after the breakpoint was used to select the 36 isolates from each group. This resulted in 12 isolates selected from those collected in 2002–2003, none from 2003–2004, 1 from 2004–2005, 1 from 2005–2006, 4 from 2006–2007, 8 from 2007–2008, 10 from 2008–2009, 9 from 2009–2010, 11 from 2010–2011, and 16 from 2011–2012.

### Selection of PCR targets

The manual BLAST searches were performed on each significant putative gene against the pre- and post-2008 isolates to determine where the genes exist on the chromosome in reference to each other. Geneious version 7 [32] was used to visualize the location of significant putative genes in relationship to each other within each isolate and comparatively across other sequenced isolates. Genes that were identified to have homologs with biologically relevant function via BLAST searches, or were co-located with other genes of interest, suggesting these genes may be in a functional unit or operon, were selected as PCR targets. Geneious software was used to confirm that primer target regions were identical across the previously sequenced *S*. Enteritidis isolates containing the genes of interest.

### PCR amplification

NORGEN Biotek Corp. bacterial genomic DNA isolation kits were used to extract the genomic DNA of the 72 non-sequenced isolates selected by simple random sampling and 2 μl of template from each isolate was included in the PCR to screen for each of the six putative genes under their optimized conditions. PCR conditions for each gene were optimized in regard to magnesium concentration and annealing temperature on isolates that had previously been sequenced [15] and verified *in silico* to contain the genes of interest. Primer mix included 2 μl 10x PCR buffer, 0.4 μl of 10 mM dNTPs, and 0.2 μl of Invitrogen Taq DNA polymerase without Mg, and 1 μl of 10 mM forward and reverse primer relevant to each targeted centroid (final volume, 18 μl). PCR was performed in an Eppendorf Mastercycler®. The cycling parameters for screening each centroid involved denaturation at 94°C for 2 min, followed by 25 cycles of heating to 94°C for 30 sec, the relevant optimized annealing temperature for 30 sec, 72°C for extension time of relevant to the product length (1 min/kbp), and a final step of 72°C for 5 min. Primer sequences and PCR conditions for each centroid are shown in Table 1.

**Table 1:** PCR specifications.

Primer	Sequence	Amplicon (bp)	Centroid Target	Magnesium concentration (μg/μl)	Annealing Temperature (°C)
pre1F	TACTTTAGCCATCGAACTGG	1353	*pre1*	1.5	60
pre1R	TCGAATCACCACGCATTGAC				
pre2F	TACAGCTCCATTAGCACAGG	1256	*pre2*	1.5	63
pre2R	TAACGGAAGGTGGTGCTGTC				
pre3F	TTGATGCTGCAGGCATTTGC	873	*pre3*	1.5	60
pre3R	TTAACTGCATCAGGGATCTC				
post1F	TTCGACAAAGATCGCATTGG	1153	*post1*	1.0	63
post1R	ACATGAAGGTCATCGATAGC				
post2F	ACTCAGTGCTTTGATGGATG	573	*post2*	1.5	60
post2R	AGCAGCATAACCTTTTTCCG				
post3F	ATACCAGAAGCCGTCGTTGG	1039	*post3*	1.0	63
post3R	AAGGCACCGACCATAGGAAC				

PCR products were separated on a 1% agarose gel stained with SYBR Safe and visualized using a UV transilluminator. The proportion of isolates in the pre- and post-2008 groups that contained the selected PCR target were compared by chi-square analysis (with a p-value adjusted for multiple comparisons by Bonferroni correction) to test for significant differences in their distribution.

### Phenotypic analysis

Initial annotation of the putative genes novel to the *S*. Enteritidis isolates collected after 2008 were found to be homologs of tetracycline-family resistance genes and arsenic utilization genes. Kirby-Bauer tests with 30 μg tetracycline were performed on the isolates which had been screened by whole genome sequencing or PCR assay to verify the predicted phenotypic differences between these strains. Colonies grown from overnight incubations were resuspended in PBS at a turbidity of 0.5 MacFarland and plated on Mueller-Hinton agar with discs impregnated with 30 μg tetracycline and concentrations of 0.04, 0.16, and 0.64 mg/ml sodium arsenate [33]. Zones of inhibition were measured after 12–18 hours of incubation at 37°C. For tetracycline antibiograms, zones with a diameter of ≥19 mm were considered susceptible, and ≤14 mm were resistant per CLSI guidelines [34]. Significant differences in the proportion of tetracycline resistant isolates from pre- and post-2008 samples were identified by chi-square analysis and differences in zone diameters of arsenic resistance were compared using Student’s t-test (p<0.05).

## Results

### Selection of temporal break point

**Fig 1:**
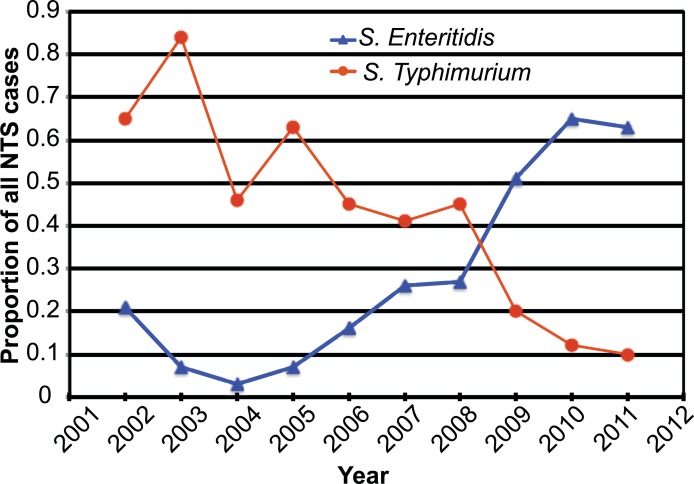
Relative proportion of iNTS cases caused by *S*. Typhimurium and *S*. Enteritidis from 2002–2012. The relative proportion of the iNTS isolates observed among pediatric admissions to the HGT as identified by Tapia et al. [19]. The observed shift in relative proportions of *S*. Enteritidis and *S*. Enteritidis occurs at approximately 2008.

**Fig 2:**
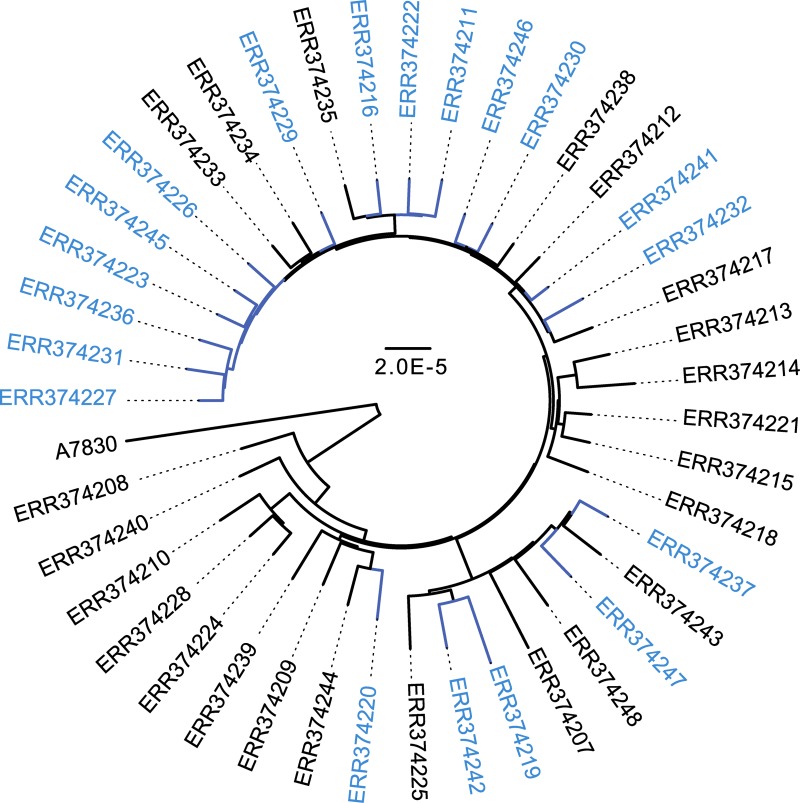
Phylogenomic analysis of pre-, during, and post-2008 *S*. Enteritidis isolates. A whole-genome phylogeny was generated for the 42 *S*. Enteritidis genomes analyzed in this study, which were collected from the HGT from July 2002 through June 2012. Isolates collected before or during 2008 are shown in black and isolates collected after 2008 are shown in blue. The tree is constructed using a maximum-likelihood phylogeny with 100 bootstrap replicates using RAxML v7.2.8. Shaded grey circles over the nodes designate bootstrap values ≥90.

While the epidemiologic analysis of iNTS by Tapia et al. [19] suggested that the increase in the relative proportion of iNTS caused by *S*. Enteritidis compared to *S*. Typhimurium occurred in 2008, the change appeared to occur gradually over time from July 2006 to June 2010 (Fig 1). Phylogenomic analysis (Fig 2) was used to determine where in this time period the breakpoint occurred, at which the novel clade was established as the predominant *S*. Enteritidis genotype in the region. The branches of the tree were manually rotated at each node to determine the potential breakpoint year to minimize the number of misclassifications (isolates collected from one side of the temporal breakpoint appear on a branch with isolates collected from the other side of the breakpoint) across the entire tree. A breakpoint of 2008 minimized the difference in misclassifications between the pre and post groups across several major branches of the phylogenetic tree (S2 Table), and therefore was accepted as the year at which the novel *S*. Enteritidis genotype was established as the dominant serovar.

### *In silico* identification of genes associated with the serovar shift

**Fig 3:**
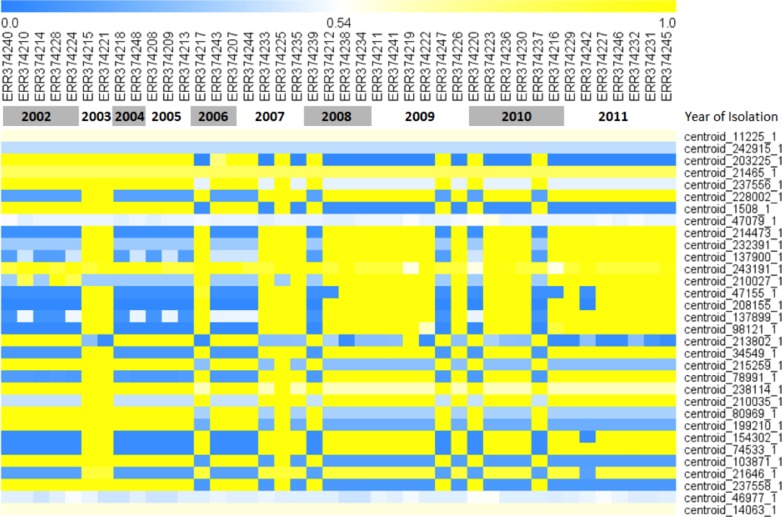
Heat map of putative genes associated with the pre- and post-2008 breakpoint. The 42 *S*. Enteritidis genomes are listed across the x-axis with putative genes significantly associated with the temporal breakpoint listed down the y-axis. BSR scores range from 1 (“present”), indicated in yellow, to 0 (“absent”), indicated in blue, for each putative gene in each sequenced isolate.

Among the 42 whole genome sequences acquired from isolates obtained from the HGT from July 2002 through July 2012, as examined by Feasey et al. [15], LS-BSR analysis identified a total of 5,883 putative genes, including 1,220 putative genes that were not completely conserved in all samples (S1 Table). After BLAST score ratios (BSRs) were calculated for each of these putative genes, 12 genes were found to be significantly more prevalent in the pre-2008 sequences and 15 significantly more prevalent in the post-2008 sequences (p<0.01 by chi-square test) (Table 2). After further manual BLAST verification, two putative genes associated with pre-2008 were identified to also be conserved in the post-2008 isolates, and were therefore removed from our analysis. These genes bridged across two contigs of the genetic sequences of post-2008 sequences and therefore were identified as false-negatives during the preliminary *in silico* analysis. A heatmap visualizing the LS-BSRs of each associated putative gene for each isolate is presented in Fig 3.

**Table 2:** Characteristics of putative genes associated with the 2008 breakpoint.

Centroid ID	Length (bp)	Temporal Breakpoint Association	Annotation	Predicted Protein Function	Reference Sequence Accession No.*
centroid_237556_1	333	PRE	-	acyl carrier protein	CP007528.1
centroid_80969_1	324	PRE	-	S-adenosylmethionine tRNA ribosyltransferase	CP007528.1
centroid_237558_1	489	PRE	-	MarR transcriptional regulator	CP007528.1
centroid_1508_1	1353	PRE	pre1	multidrug tranporter (MarR)	CP007528.1
centroid_103871_1	1242	PRE	-	12-TMS multidrug efflux protein homolog	CP007528.1
centroid_203225_1	1256	PRE	pre2	PhoPQ-regulated protein	CP007528.1
centroid_199210_1	1287	PRE	-	putative transport protein	CP007528.1
centroid_215259_1	873	PRE	pre3	L-galactonate dehydrogenase	CP007528.1
centroid_238114_1	2613	PRE	-	fimbrial Protein	CP007528.1
centroid_213802_1	1149	PRE	-	Lactaldehyde reductase	CP007528.1
centroid_21646_1	354	POST	-	Hypothetical protein	AP010961.1
centroid_154302_1	807	POST	-	Hypothetical protein	AP010961.1
centroid_208155_1	1398	POST	-	putative transposase/Not Found	AP010961.1
centroid_98121_1	1209	POST	-	IS4 transposase	AP010961.1
centroid_47155_1	708	POST	-	Hypothetical protein	AP010961.1
centroid_228002_1	255	POST	-	tn10 Tetc protein transposon	AP010961.1
centroid_137900_1	1153	POST	post1	Class B Tetracycline resistance protein (TetA)	AP010961.1
centroid_137899_1	602	POST	-	Ars R family transcriptional regulator	AP010961.1
centroid_214473_1	624	POST	post2	Ars R family transcriptional regulator	AP010961.1
centroid_34549_1	573	POST	-	transposase, ArsR family transcriptional regulator	AP010961.1
centroid_78991_1	321	POST	-	transposase, ArsR family transcriptional regulator	AP010961.1
centroid_210035_1	1039	POST	post3	Sodium-glutamate symporter	AP010961.1
centroid_232391_1	105	POST	-	IS4 transposase, sodium-glutamate symporter	AP010961.1
centroid_74533_1	1269	POST	-	Hypothetical protein	AP010961.1
centroid_210027_1	276	POST	-	Hypothetical protein	AP010961.1

* Reference Sequence Accession numbers indicate the closest match in GenBank to the isolated *S*. Enteritidis centroid sequences

### Selection of PCR screening targets

Targets for PCR screening were selected based on genes with functional annotation which could be phenotypically validated and based on the co-location of the genes of interest. Among the isolates collected before or during 2008, *S*. Enteritidis genes encoding oxidoreductases, a multidrug transcription regulator (*marR*) and efflux proteins, and putative transport and membrane export proteins were identified (Table 2). Most of the genes associated with the post-2008 time period were not identified among previously sequenced *S*. Enteritidis available in the NCBI database. However, homologs of insertional transposases, *arsR* family arsenic-dependent transcriptional regulators, sodium-dependent glutamate permeases, and tetracycline resistance/efflux proteins (*tetA*) were identified (Table 1). These homologs shared greater than 95% coverage and nucleotide identity with the putative genes of interest in *S*. Enteritidis isolates (with the exception of centroid_21646_1, for which no homolog was identified through BLAST). Of the 25 investigated genes, seven (7/25, 30%) have no functional homolog and are considered hypothetical proteins.

**Fig 4:**
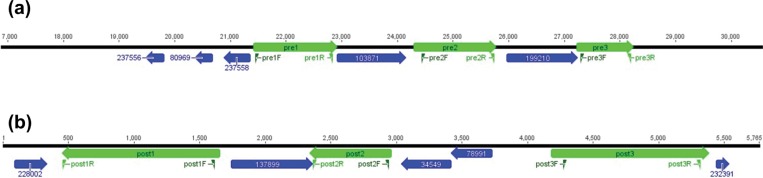
Gene mapping. (a) Map of the genes associated with the pre-2008 time period (b) Map of the genes associated with the post-2008 time period; genes selected for PCR screening are indicated in green (*pre1*, *pre2*, *pre3* and *post1*, *post2*, *post3*), other genes significantly associated with the time threshold are shown in blue.

Mapping of the pre- and post-2008 genes to isolates sequenced for this study suggested that many of the putative genes of interest appear to be located in close proximity, or on the same genomic contig with consistent arrangement and orientation (Fig 4). This suggests that these isolates may have acquired a genomic island or other mobile element that allowed expansion into this niche. Definitive identification of these mobile elements will require further study.

### PCR screening

Primers were designed to target each centroid of interest (Table 2 and Fig 4). The results of PCR screening the six selected genes across the 72 non-sequenced isolates exhibited a similar distribution of genes as was identified through the bioinformatic analysis (Table 3). Each of the three genes associated with the pre-2008 time period were identified in significantly more of the isolates collected during or before 2008, compared to those collected after (p = 0.0008, 0.0078, and 0.0047, respectively), suggesting that these genes were lost in the post-2008 isolates. Similarly, each of the three genes associated with the post-2008 time period were identified in significantly more of the isolates collected during that time, compared to isolates collected in the earlier years (p<0.0001, 0.0008, and <0.0001, respectively). This represents an acquisition of these genes in the post-2008 isolates.

**Table 3:** PCR screening results.

Putative Gene	No. of positive isolates, pre-2008 (n = 36)	No. of positive isolates, post-2008 (n = 36)	p-value*
*pre1*	21	7	0.0008
*pre2*	19	8	0.0078
*pre3*	26	14	0.0047
*post1*	6	25	<0.0001
*post2*	15	29	0.0008
*post3*	6	27	<0.0001

* calculated by chi-square test

### Phenotypic results

To validate the *in silico* results, phenotypes for tetracycline and arsenic resistance were assessed by resistance assays (Table 4). No statistically significant difference was observed in the arsenic resistance among the isolates at any of the three concentrations tested. Tetracycline resistance was identified to be highly associated with the 2008 threshold, with 53% more isolates resistant to tetracycline after 2008 than during or prior to 2008 (p = 0.0008). In comparing these results, the presence of the tetracycline resistance gene screened by PCR (*post1*) is strongly associated with phenotypic tetracycline resistance as observed by antibiogram assay (p = 0.0016).

**Table 4:** Resistance assay results.

**Arsenic Resistance**	**Zone of Inhibition, mean diameter (mm)**	**Zone of Inhibition, mean diameter (mm)**	p-value**
4 μg/μl Concentration	9.03	9.28	0.5571
16 μg/μl Concentration	11.89	11.78	0.7281
64 μg/μl Concentration	15.06	14.69	0.3670
**Phenotype**	**No. of positive isolates, pre-2008** **(n = 36)**	**No. of positive isolates, post-2008** **(n = 36)**	p-value*
Tetracycline Resistance	19	32	0.0008

* calculated by chi-square test

** calculated by t-test

## Discussion

*S*. Typhimurium was initially identified as the predominant serovar of iNTS in most, albeit not all, regions in sub-Saharan Africa [12–14]. We identified that 2008 marked a breakpoint in the relative proportions of iNTS caused by *S*. Enteritidis versus *S*. Typhimurium in Bamako, Mali, collected from 2002 to 2012. Additionally, we identified that the newly circulating clade of *S*. Enteritidis exhibits greater levels of tetracycline resistance than previous strains of *S*. Enteritidis. Our temporal breakpoint agrees with the epidemiologic data from Tapia et al. [19] and the phylogenomic analysis by Feasey et al. [15]. Our finding that *S*. Enteritidis represents an emerging threat of invasive bacterial disease in Bamako, Mali is mirrored by similar findings from other parts of sub-Saharan Africa [14]. Additionally, surveillance data from 2014 through 2017 with complete serovar determination support this continued change in prevalence as there were 28, 15, 16 and 14 cases of invasive *Salmonella* disease that occurred, respectively during 2014, 2015, 2016 and 2017. During this four-year period *S*. Enteritidis was the most commonly identified organsim (*S*. Enteritidis (N = 28), *S*. Typhimurium (N = 12), *S*. Typhi [11] and *S*. Dublin (N = 9) accounted for 63 of the isolates from the 73 cases of invasive disease). Importantly *S*. Enteritidis is associated with a significantly greater case fatality rate than *S*. Typhimurium in all pediatric age groups [12].

The current study markedly expands the number of genome sequences of *S*. Enteritidis from iNTS cases available in public databases. Furthermore, our study describes an approach to examining these genomes, combining whole genome sequencing and large-scale BLAST score ratios (LS-BSRs) [26]. The use of the LS-BSR method allows investigation of major genetic changes across the genome by identifying and comparing putative gene content among a collection of genome sequences. In an under researched clade of a *Salmonella* serovar, such a broad sweeping approach was integral to identifying phylogenomic changes over time.

The underlying cause of the shift in serovar predominance resulting in the emergence of *S*. Enteritidis as the leading cause of iNTS disease, has yet to be fully explained, and we anticipate that the bacterial component of this observation is only part of the larger infection picture. We identified that the recently emerged circulating clade of *S*. Enteritidis exhibits greater levels of tetracycline resistance than previous strains of *S*. Enteritidis. This phenotype may provide the serovar with an evolutionary advantage over *S*. Typhimurium, contributing to the shift wherein *S*. Enteritidis became the predominant serovar causing iNTS disease in Bamako [19]. Host-mediated hemolysis caused by malaria impairs resistance against iNTS infections [35,36]. Resistance to doxycycline, a long-acting member of the tetracycline family of drugs that can be used for prophylaxis and treatment of malaria [37,38], could theoretically convey an evolutionary advantage to *S*. Enteritidis by allowing it to survive during malaria prophylaxis or treatment. However, in the Mali context this is implausible because doxycycline and other tetracyclines are rarely used for chemoprophylaxis or treatment of malaria, particularly in infants and young children. However, tetracyclines and other antibiotics are sometimes inserted into potions used to treat fever by traditional healers, but the relative frequency of this practice is not easily quantified. Thus, another explanation must be sought for acquisition of tetracycline genes conveying an increased fitness. Expression of the *tetA* resistance gene has also been associated with increased influx and accumulation of toxic heavy metal salts in *E*. *coli* [33], which may help explain the simultaneous acquisition of this gene with the *arsR* regulator in the post-2008 isolates.

The arsenic resistance assay performed in this study was selected as a screening method to verify the *in silico* findings, but the inconclusive results do not negate the bioinformatic analysis which clearly suggested that the acquisition of an *arsR* regulator gene is associated with the 2008 shift in prevalence of *S*. Enteritidis over *S*. Typhimurium. It is possible that our measure of arsenic resistance does not adequately assess the potential phenotypic and global transcriptomic changes generated by the *arsR* gene product. It is also possible that the *arsR* homolog gene product regulates other phenotypes that have not been examined in this study. Future studies should investigate alternative metabolism assays, arsenic utilization assays, and genetic knockouts to more thoroughly investigate the possible evolutionary advantages granted by the acquisition of this gene by *S*. Enteritidis.

By identifying putative genes at a 90% nucleotide identity level, major genetic changes were identified that appear to be novel to the *S*. Enteritidis serovar. Future studies should further investigate the difference identified to determine the precise nature of the genetic acquisitions and search for additional significant changes at the single nucleotide level. Additional research involving genetic knock outs and complementation will be necessary to fully assess the impact of each potential gene on the phenotype of the organism and to formally identify which phenotypic and genotypic features should be examined through GWAS. Additionally, the shift in NTS serovars was identified to be associated with a specific year, but it is possible that the evolution of infectious disease may occur as a more gradual phenomenon that cannot truly be dichotomized to a single year. As such, our 2008 cut point to identify the novel phylogenomic clade could be subject to misclassification bias. We have attempted to minimize this misclassification by minimizing the amount of differential misclassification between pre- and post-2008 groups, thus biasing our results towards the null.

The concurrent acquisition of the putative novel genes, and the increase in relative importance of the serovar in causing invasive bacterial disease suggests that the genetic changes may provide an evolutionary advantage to *S*. Enteritidis over *S*. Typhimurium. This appears to have resulted in *S*. Enteritidis losing minor enzymes and a putative multidrug transporter in order to gain resistance to tetracycline family antibiotics and an arsenic responsive transcriptional regulator. These data provide a potentially important clue that can be pursued through future research to characterize the competitive advantages and to confirm that no other change in *S*. Typhimurium is responsible for the decline of that serovar relative to *S*. Enteritidis. These findings will help illuminate the public health effects of these evolutionary changes to the serovar and shed more light on the evolutionary pressures on *S*. Enteritidis. Further analyses of the functional effects of this documented change in antibiotic resistance or other phenotypes in the natural environment can help generate novel hypotheses of natural reservoirs of NTS and help elucidate how the *S*. Enteritidis serovar competes with *S*. Typhimurium within the human host or in an environmental reservoir or vehicle of transmission.

## Supporting information

S1 Table Large Scale BLAST Score Ratios (LS-BSR) data. LS-BSR scores of each of the all the putative genes identified among the 42 sequenced *S*. Enteritidis isolates, with ENA Run reference numbers and year of clinical isolation (based on the June-July seasonal year).
(XLSX)

S2 Table Distribution of misclassified isolates by year. The proportion of misclassified isolates distributed across the branches of the tree using each year of the study as a potential breakpoint for the serovar shift between *S*. Typhimurium and *S*. Enteritidis. Isolates were considered misclassified when they were collected from one half of the chronology but appeared in a branch that consisted mainly of isolates from the other half of the chronology. Branches enumerated from the root outward and were manually rearranged to minimize the number of misclassifications across the tree for each breakpoint. A breakpoint of 2008 provides the lowest minimal misclassifications across both the pre- and post-breakpoint groups, indicated by (*).
(XLSX)
